# Salicylhydroxamic acid as an electro-responsive and switchable adhesive molecule

**DOI:** 10.1039/d5sc07881d

**Published:** 2026-04-02

**Authors:** Kan Wang, Vedika Khare, Abhilash Arjan Das, Fatemeh Razaviamri, Bruce P. Lee

**Affiliations:** a Biomedical Engineering Department, Michigan Technological University 1400 Townsend Drive Houghton Michigan 49931 USA bplee@mtu.edu

## Abstract

This study investigated salicylhydroxamic acid (SHAM) as a new electro-responsive, switchable adhesive molecule for wet adhesive contact. A SHAM-containing polymer was synthesized and evaluated using a customized Johnson–Kendall–Roberts (JKR) contact mechanics setup, where a titanium (Ti) hemisphere simultaneously functioned as the contact substrate and cathodic electrode. Application of electrical potential (0.5–2 V) for 30 s or less reduced the work of adhesion by 84%, while subsequent incubation in a pH 5 buffer restored the original work of adhesion value. Unlike the catechol-containing polymer, which underwent irreversible oxidation to quinone, SHAM demonstrated fully reversible switching without the need for a protective group. Cyclic voltammetry and electrochemical impedance spectroscopy confirmed that both SHAM- and catechol-containing polymers exhibited comparable electrical conductivity, ensuring that the observed differences in adhesion behaviors stemmed from their intrinsic molecular properties. The combination of UV-vis, Fourier transform infrared, and X-ray photoelectron spectroscopy experiments confirmed that the switching mechanism arose from protonation and deprotonation of SHAM, which regulated SHAM's ability to bond to the Ti surface. Conversely, catechol irreversibly oxidized to the poorly adhesive quinone form and failed to regain its initial adhesive properties. SHAM exhibited electro-responsive interfacial bonding capability to a metallic surface under wet conditions and is potentially suitable for designing new switchable adhesives.

## Abstract 2

Salicylhydroxamic acid exhibits electro-responsiveness and switchable interfacial bonding to metallic surfaces under wet conditions without the need for protecting groups.

## Graphical abstract

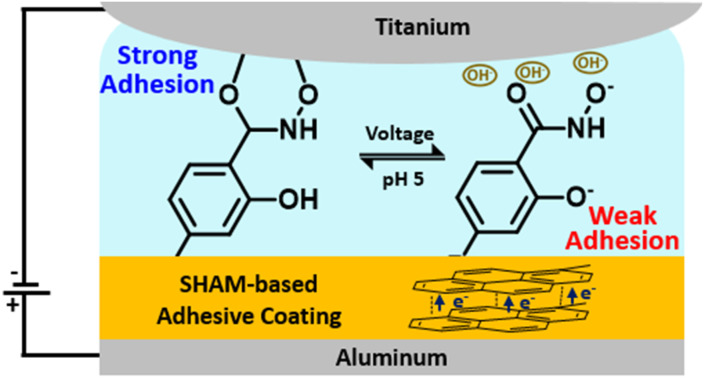

## 1. Introduction

Switchable adhesives with tunable, on-demand adhesion have attracted considerable interest for their potential in advancing research fields ranging from biomedical devices to robotic manipulation and wearable electronics.^1–4^ By enabling reversible bonding and detachment under external stimuli (*e.g.*, light, temperature, pH, and electric fields), these materials offer a sustainable strategy to reduce material waste, streamline assembly and disassembly, and minimize substrate damage during removal.^5,6^ However, many sources of external stimuli have limitations and are not practical. For example, light-responsive adhesives are limited to adhesive joints formed using transparent substrates,^7^ while thermo-responsive adhesives may require excessive heat leading to surface damage.^8^ pH-triggered adhesion systems are inherently diffusion limited, with a slow switching rate between adhesive and non-adhesive states.^9^ In contrast, electrical stimulation offers superior controllability, ease of operation, and seamless integration with electronic devices.^3,10,11^ Yet, most electro-responsive adhesives still require high voltage levels, making it challenging to balance strong adhesion with low-energy debonding.^12^ This challenge highlights the critical need for electro-responsive adhesives that operate effectively at low voltages.

One general strategy in designing a switchable adhesive is the incorporation of stimuli-responsive adhesive molecules into an adhesive polymer matrix.^13^ A well-established example is the redox active catechol, which is one of the main adhesive molecules found in mussel foot proteins.^14–16^ The adhesive properties of catechol are highly dependent on its oxidation state, where the reduced form of catechol demonstrates strong adhesion but is significantly weakened when oxidized into quinone.^17^ Electrochemical oxidation of catechol can reduce its adhesion strength by up to 96%.^18^ However, quinone is highly reactive and prone to irreversible crosslinking, which compromises reversibility.^19^ To mitigate this limitation, we previously introduced boronic acid as a temporary protecting group to suppress irreversible catechol oxidation.^20^ Catechol–boronic acid adhesives could be detached from metal substrates under an applied electrical potential and partially regenerated through incubation in an acidic buffer.^21^ However, it is difficult to completely dissociate the catechol–boronate complex to fully regain the initial adhesive properties.^9^ As such, there remains a pressing need for alternative adhesive molecules that combine strong and reversible adhesion that is responsive to applied electricity.

**Fig. 1:**
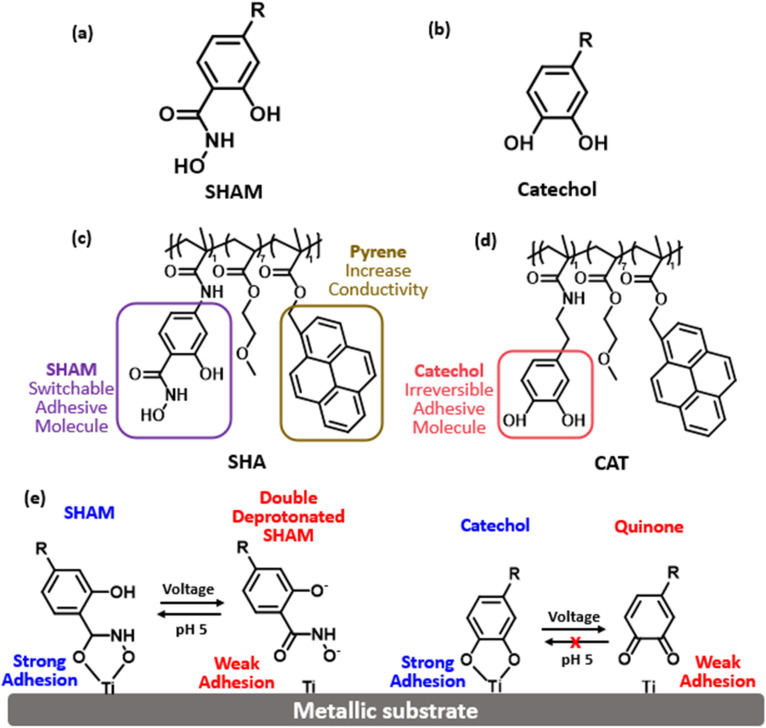
Chemical structures of (a) SHAM, (b) catechol, (c) SHAM-based polymer (SHA) and (d) catechol-based polymer (CAT). (e) Electrochemical deactivation of SHAM and catechol. SHAM could be reactivated with a mildly acidic solution, while catechol becomes irreversibly oxidized without a protecting group.

Salicylhydroxamic acid (SHAM) has recently emerged as a promising candidate for a switchable adhesive molecule and exhibits pH-responsive adhesion to wet surfaces (Fig. 1).^22^ When incorporated into structural adhesives, SHAM achieved lap shear strengths that were significantly higher than those of catechol-based adhesives and a commercial epoxy glue.^23^ Unlike catechol, SHAM features both a hydroxamic acid group and a phenolic hydroxyl group. Hydroxamic acids, bearing bidentate (O,O) donor sets, are well known for strong metal chelation.^24,25^ For example, SHAM adhesion to titanium depends on its protonation state and can be reversibly modulated by pH.^22^ Under basic conditions, deprotonated SHAM exhibits reduced adhesion, whereas protonation under acidic conditions restores full adhesion. In a typical electrochemical reaction, local accumulation of hydroxide (OH^−^) and protons (H^+^) near the cathode and anode, respectively, creates a pH gradient,^26,27^ which could potentially be utilized to tune the adhesive properties of SHAM.

In this study, we investigated the feasibility of SHAM to function as an electro-responsive, switchable adhesive molecule without the use of a protecting group. We synthesized a SHAM-based adhesive consisting of a methoxyethyl methacrylate (MEA) backbone and a conductive pyrene moiety (Fig. 1c). The adhesive was coated onto aluminum (Al) discs (serving as the anode), and the adhesive properties of the SHAM-containing polymer were characterized using Johnson–Kendall–Roberts (JKR) contact mechanics testing using a titanium (Ti) hemisphere (cathode) as the test substrate. The effect of applied electrical potential and exposure time on the adhesive properties of SHAM-based adhesive (SHA) was determined and benchmarked against a catechol-based polymer (CAT). In addition, UV-vis spectroscopy, Fourier transform infrared (FTIR) spectroscopy, and X-ray photoelectron spectroscopy (XPS) were employed to characterize the redox state of adhesive moieties following electrical treatment, while cyclic voltammetry (CV) and electrochemical impedance spectroscopy (EIS) were used to assess the conductivity of the adhesive coatings.

## 2. Materials and methods

### 2.1. Materials

MEA, 1-pyrenylmethyl methacrylate (PyMA), 1-methyl-2-pyrrolidinone (NMP), polyvinylidene fluoride (PVDF), silicon oil, dimethyl sulfoxide-d_6_ (DMSO-d_6_), hydrochloric acid (HCl), sodium hydroxide (NaOH), and sodium chloride (NaCl) were purchased from Sigma-Aldrich (St. Louis, MO). Dimethylformamide (DMF) and diethyl ether were purchased from Fisher Scientific (Fair Lawn, NJ). 2,2′-Azobis(isobutyronitrile) (AIBN) was purchased from Wako Pure Chemical Industries, Ltd (Osaka, Japan). The Ti ball (1/4″ diameter), 303 stainless steel (SS) rod (1/2″ diameter), Al rods (1/2″ and 3/8″ diameters), copper wire, and polytetrafluoroethylene (PTFE) (3/8″ diameter) were purchased from McMaster-Carr (Elmhurst, IL). SS and Al rods with a diameter of 1/2″ were machined into hemispheres with a diameter of 5.9 mm. The Al rod with a diameter of 3/8″ was trimmed into Al plates using an 8026J Clausing/Colchester Manual Engine Lathe (Clausing Industrial; Kalamazoo, MI). The electrolyte was prepared using deionized (DI) water and 0.1 M NaCl, and the pH was adjusted to 7.4. Two adhesive monomers, *N*,2-dihydroxy-4-methacrylamidobenzamide (DHMAAB) and dopamine methacrylamide (DMA), were synthesized according to previously published protocols.^18,22^

### 2.2. Preparation and characterization of adhesive polymers

The adhesive polymers were prepared by thermo-initiated polymerization using AIBN as the initiator (Fig. S1). To prepare the SHAM-based polymer (SHA), 10 mmol MEA (1.29 mL), 1 mmol DHMAAB (236 mg), 1 mmol PyMA (300 mg), and 0.3 mmol AIBN (49 mg) were dissolved in 10 mL DMF. The solution was stirred at 500 rpm and heated at 70 °C in a silicon oil bath under a nitrogen atmosphere overnight. The resulting polymer was precipitated in diethyl ether, filtered, and vacuum-dried for 24 hours. For comparison, a catechol-based copolymer (CAT) was synthesized using DMA instead of DHMAAB with a monomer ratio of 7 : 1 : 1 : 0.3. The chemical compositions of two adhesive polymers were investigated by proton nuclear magnetic resonance (^1^H NMR) spectroscopy using an Ascend 500 MHz spectrometer (Bruker Corporation; Billerica, MA). The weight-average molecular weight (*M̄*_w_), number-average molecular weight (*M̄*_n_), and polydispersity index (PDI) of the adhesive polymers were determined by gel permeation chromatography (GPC) using a Shimadzu Nexera HPLC system (Kyoto, Japan) equipped with a Shodex OHpak LB-803 column, a UV detector (SPD-40, Shimadzu), a refractive index detector (RID-20A, Shimadzu), and a multi-angle light scattering detector (miniDAWN, Wyatt). Polymer samples were dissolved in HPLC-grade DMSO at a concentration of 5 mg mL^−1^ and eluted at 0.5 mL min^−1^ using HPLC-grade DMF as the mobile phase at 40 °C.

The absorption spectra of 0.2 mmol L^−1^ DHMAAB and DMA aqueous solutions at different pH levels were examined using UV-vis spectroscopy (LAMBDA 35, PerkinElmer, MA). Spectra were first recorded after dissolving the monomers in 0.1 M NaCl solution (electrolyte) containing 0.2 mM adhesive monomer. To mimic the alkaline environment detected using a pH strip during electrochemical deactivation, a small aliquot of 1 M NaOH solution (pH 14) was added to the cuvette to increase the solution pH to ∼9. Subsequently, a small aliquot of 1 M HCl solution (pH 0) was introduced to reduce the solution pH to ∼5, reproducing the recovery conditions and enabling evaluation of the reversibility of the adhesive molecules.

### 2.3. Adhesive-coating preparation and characterization

The adhesive precursor solution was prepared by dissolving the adhesive polymer and PVDF in NMP at a weight ratio of 9 : 1 at final polymer concentrations of 100 and 11 mg mL^−1^, respectively. 15 µL of precursor solution was pipetted onto an Al disc (3/8″ diameter) and left in a fume hood overnight to allow complete evaporation of NMP (Fig. S2). The coated samples were vacuum dried and analyzed using FTIR spectroscopy (IRTracer-100, Shimadzu Corporation; Kyoto, Japan). The morphology of the coating was visualized using a Hitachi S-4700 field emission scanning electron microscope (FE-SEM, Tokyo, Japan).

To evaluate the electrochemical properties of the adhesive, 2 µL of precursor solution was deposited onto the working electrode (WE) of a commercial gold-coated interdigitated electrode (IDE, Medetronix Labs Pvt Ltd, Uttar Pradesh, India) while leaving the counter electrode (CE) and reference electrode (RE) uncoated (Fig. S3). The coating was left to dry in a fume hood overnight. This process yielded a thin adhesive film with an area of approximately 7 mm^2^ on the detection zone. Electrochemical measurements were performed in 10 mL of 0.1 M NaCl solution (pH 7.4), sufficient to submerge all electrodes. CV was carried out by sweeping the potential between −2 V and +2 V at a scan rate of 0.1 V s^−1^. EIS was conducted using the same adhesive-coated IDE and electrolyte, with a perturbation amplitude of 5 mV s^−1^ over a frequency range of 0.1–10^6^ Hz. The charge transfer resistance (*R*_c_) of the adhesive coating was extracted from the EIS spectrum. In Nyquist plots, the high-frequency intercept on the *X* axis corresponds to the solution resistance, while the second *X*-intercept, obtained by extending the semicircle at lower frequencies, reflects the combined resistance of ion and charge transfer. *R*_c_ was determined as the difference between these two *X*-intercepts.^21^ Both CV and EIS were performed using a VersaSTAT3 Potentiostat Galvanostat (10–240 VAC; 50/60 Hz; 250 VA Max) from AMETEK Scientific Instruments (Berwyn, PA).

### 2.4. JKR contact mechanics tests

**Fig. 2:**
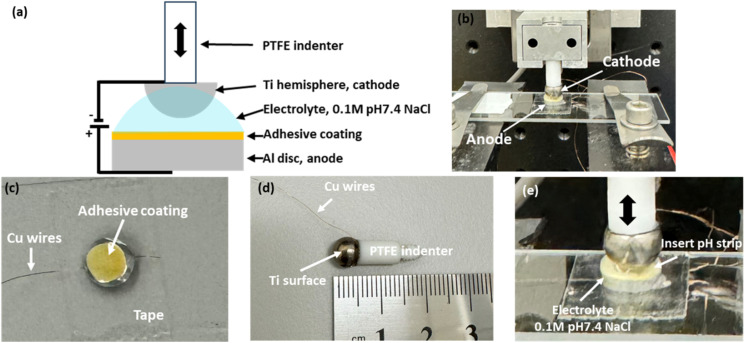
(a) Schematic representation and (b) photograph of the custom-built JKR setup used for adhesion testing with the application of electricity. Close views of the (c) anode, (d) cathode, and (e) interface of the adhesion testing.

The JKR testing device consisted of a custom-built PTFE indenter, a 10 g load cell (ALS-06, Transducer Techniques), and a high-resolution miniature linear stage stepper motor (MFA-PPD, Newport) (Fig. 2). A Ti hemisphere was attached to the indenter with a copper wire embedded at the interface (Fig. 2d). The adhesive surface was wetted with 15 µL of 0.1 M NaCl solution at pH 5 (Fig. 2e). During contact testing, the Ti hemisphere was brought into contact with the adhesive-coated surface at a speed of 1 µm s^−1^ until a maximum preload of 10 mN. Then, the Ti hemisphere was retracted at the same speed. Based on a JKR model that illustrates a rigid spherical surface acting on a thin coating over a rigid substrate, the work of adhesion (*W*_adh_), equal to the critical energy release rate (*G*_c_), was calculated from the maximum adhesion force (*F*_max_) and the radius (*R*) of the Ti tip as follows:^28–30^1
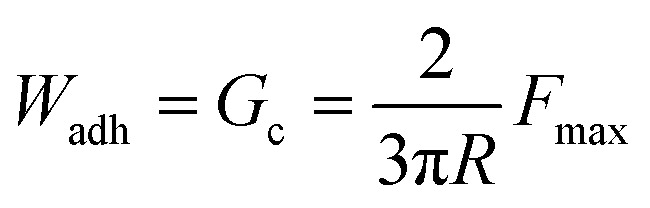


The initial adhesion, measured without any applied voltage, was recorded as the virgin adhesive properties. To examine the effect of electrical potential, the pH 5 NaCl solution was replaced with a pH 7.4 NaCl solution. The Ti hemisphere was immersed in the solution and brought close to the adhesive without contacting the adhesive. The Ti hemisphere, electrolyte, adhesive-coated Al disc, and a Keithley 2460 Source Meter formed a complete electrical circuit. Voltages between 0.1 and 2 V were applied for 15–45 seconds to deactivate the adhesive. After voltage application, the Ti hemisphere was brought into contact with the surface to determine the *F*_max_ as described above. To recover the adhesive properties of SHA, deactivated samples were incubated in 0.1 M NaCl solution at pH 5 for 30 minutes before performing another adhesive contact. Adhesive reversibility was assessed over five cycles of deactivation (1 V for 30 s) and recovery (30 min). JKR experiments were also performed using SS and Al hemispheres to determine the interfacial bonding behavior of SHA to these metals and the ability of these metals to function as an electrode to deactivate the adhesive properties of SHA.

### 2.5. XPS

15 µL of 100 mg mL^−1^ adhesive polymer in NMP was applied onto Al discs (diameter: 6.33 mm) and dried in a fume hood for 24 hours. To deactivate the adhesive, 15 µL of 0.1 M NaCl (pH 7.5) was added to the adhesive surface, a Ti disc (diameter: 6.33 mm; cathode) was placed near the adhesive coating without making contact, and a voltage of 1 V was applied for a duration of 30 seconds using the Al disc as the anode. Reactivation was performed by incubating the coated discs in a pH 5 0.1 M NaCl solution for 30 minutes. The samples were vacuum dried before XPS measurements were conducted.

XPS spectra were obtained utilizing a PHI 5800 XPS system (Physical Electronics, Chanhassen, MN, USA) that features a dual Mg/Al X-ray source. Measurements were conducted utilizing Mg Kα radiation, with a photon energy of 1253.6 eV. Survey spectra were obtained using a pass energy of 187.85 eV, a step size of 0.8 eV, and a dwell time of 20 ms per step in a binding energy range of 0–1175 eV. High-resolution spectra of the C 1s and O 1s regions were acquired with a pass energy of 23.50 eV, a step size of 0.1 eV, and a dwell time of 100 ms for each step. Charge neutralization was implemented during the spectral acquisition process. All spectra were processed with CasaXPS software (Casa Software Ltd, Teignmouth, UK). Calibration of the binding energy was conducted using the adventitious C–C/C=C peak at 284.8 eV as a reference. Peak fitting utilized Shirley background subtraction and a combination of Gaussian and Lorentzian peak shapes. The peak areas were normalized to the total envelope area, and the component fractions are reported as relative area percentages.

### 2.6. Statistical analysis

One-way analysis of variance (one-way ANOVA) with Tukey's method was used for comparing multiple groups, using a *p*-value of 0.05.

## 3. Results and discussion

### 3.1. Preparation of adhesive polymers and adhesive-coated Al discs

Adhesive polymers were synthesized using an MEA backbone, adhesive monomers (DHMAAB for SHA and DMA for CAT), and the conductive monomer PyMA *via* thermally initiated free radical polymerization (Fig. S1). The resulting polymers contained SHAM or catechol side chains, enabling switchable adhesion to metal surfaces. PyMA was incorporated to enhance conductivity through π–π stacking interactions from pyrene groups, thereby enhancing electron mobility and the overall conductivity.^21^ The chemical compositions of the copolymers were determined by ^1^H NMR spectroscopy (Fig. S4 and S5). Characteristic peaks were observed for MEA (3.2–4.1 pm), the aromatic ring of catechol (6.4–6.7 ppm), the aromatic ring of SHAM (7.1–7.7 ppm), and pyrene (7.5–8.5 ppm). Integration of these peaks indicated the molar ratios of MEA : adhesive monomer (DHMAAB or DMA) : PyMA to be 7 : 1 : 1. The polymers were designated as SHA and CAT for SHAM- and catechol-containing copolymers, respectively. Based on GPC, SHA exhibited a significantly higher *M̄*_n_ (217 kDa) when compared to that of CAT (16.3 kDa) (Table S1). The lower molecular weight of CAT may be due to the partial inhibition of the free-radical polymerization by the unprotected catechol.^31^ Nevertheless, the SHAM and catechol contents in the two copolymers were both found to be ∼11 mol% based on ^1^H NMR spectra, and the difference in the MW will not affect the adhesion values measured based on JKR contact mechanics testing.^32^

### 3.2. CV and EIS analyses

**Fig. 3:**
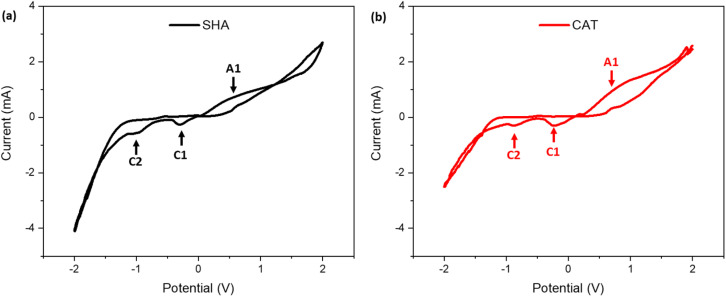
Cyclic voltammograms of (a) SHA- and (b) CAT-coated IDEs in 0.1 M NaCl solution (pH 7.4) at a scan rate of 100 mV s^−1^.

CV was performed over a potential range of ±2 V to investigate the redox behavior of the copolymers. SHA exhibited an anodic peak at +0.54 V, while CAT displayed a peak at +0.95 V, both corresponding to oxidation of their respective functional groups (Fig. 3 and Table S2). The lower anodic peak potential for SHAM indicated that it is easier to oxidize than catechol.^33^ In contrast, SHA exhibited two cathodic peaks at −0.31 V and −0.98 V, while CAT showed peaks at −0.22 V and −0.85 V, suggesting that catechol undergoes reduction more readily than SHAM.^33^ The peak current intensities of SHA and CAT were comparable, reflecting similar charge-transfer capacity. This is potentially due to similar PyMA content (∼11 mol% based on ^1^H NMR spectra) in both copolymers, which contributed to electron conductivity.^21^

To further investigate the electronic properties of the copolymers, EIS was conducted on adhesive-coated IDEs (Fig. S6). The Nyquist plots displayed a partial semicircle in the high-frequency region followed by a Warburg diffusion-related linear curve at lower frequencies.^34,35^ Although our recorded spectra did not display a complete semicircle due to our instrumental limitations, reliable *R*_c_ values were obtained through equivalent circuit fitting using the ZView software. *R*_c_, which represents the electron transport across the electrode–electrolyte interface,^34^ was determined to be 34.5 ± 4.5 Ω mm^−2^ and 31.8 ± 1.8 Ω mm^−2^ for SHA and CAT, respectively. These values indicate that both copolymers exhibit comparable electrical conductivity, which minimizes the influence of polymer resistance on electrical stimulation. Thus, subsequent adhesion experiments primarily reflect the intrinsic adhesive performance of SHAM *versus* catechol, rather than the confounding differences in electronic properties.

### 3.3. Effect of exposure time on electricity

The adhesive precursor solution was coated directly onto Al discs using PVDF as a binder to form a cohesive film on the Al surface. This procedure is consistent with methods used in battery electrodes, wearable electronics, and advanced coatings.^36^ The coating thickness was measured by FE-SEM to be approximately 60 µm for both copolymers (Fig. S7). The coating thickness was deliberately minimized to reduce the electrical resistance, consistent with the reported inverse relationship between the membrane resistance and thickness.^37^ The interfacial bonding behavior and electro-responsiveness of the coatings were evaluated using JKR contact mechanics tests (Fig. 2). A Ti hemisphere was employed both as the conductive electrode and the contact substrate. Ti serves as a representative model surface as both SHAM and catechol have previously been demonstrated to strong interfacial bonding to Ti.^23^ The Al disc serves as the counter electrode due to its high conductivity and inherent corrosion resistance.^38,39^ 0.1 M NaCl was used as the interfacial electrolyte to complete the circuit between electrodes, power supply, and conductive coating while simultaneously serving as the aqueous medium for wet adhesion contact.

**Fig. 4:**
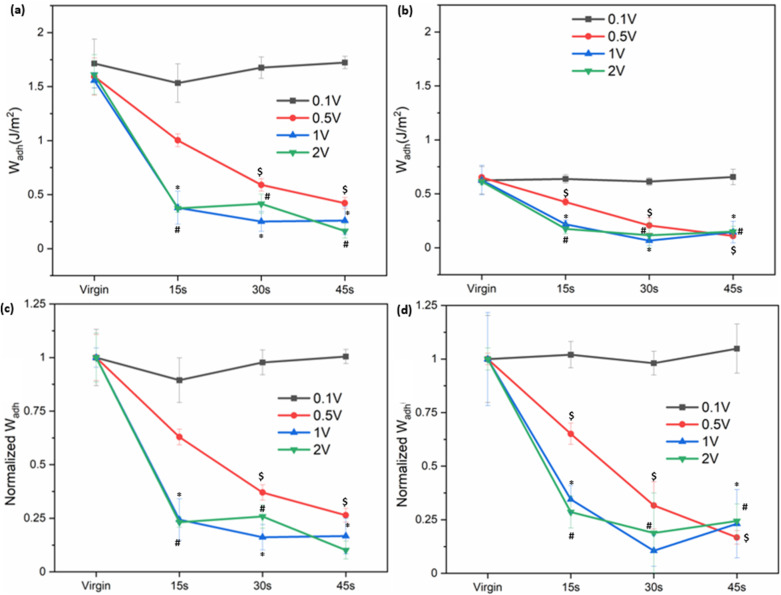
*W*_adh_ of (a) SHA and (b) CAT coating exposed to 0.1 V–2 V for different amounts of time in the presence of an electrolyte of pH 7.4. (c and d) *W*_adh_ values normalized to their average virgin adhesion values for SHA and CAT, respectively. ^%, $, *, #^*p* < 0.05 when compared to the virgin adhesive properties for 0.1, 0.5, 1, and 2 V, respectively.

Prior to electrical stimulation, the virgin *W*_adh_ values of SHA and CAT coatings were determined to be 1.59 ± 0.17 and 0.62 ± 0.12 J m^−2^, respectively (Fig. 4 and S8). The higher *W*_adh_ value of SHA indicates superior adhesion relative to CAT. Application of 0.1 V for up to 45 s did not significantly alter the calculated *W*_adh_ values, indicating that such a low potential was insufficient to deactivate adhesion in either polymer. At higher voltage levels, *W*_adh_ decreased progressively with increasing applied potential. For example, applying electrical potentials of 0.5, 1, and 2 V for 15 s reduced the *W*_adh_ values of SHA by approximately 37%, 76% and 77%, respectively, relative to the virgin state. Similarly, *W*_adh_ values also decreased proportionally with increasing exposure time for both adhesives. These results indicated that the higher applied voltage and longer exposure time to applied electricity accelerated adhesion deactivation.

JKR experiments were also performed using SS and Al hemispheres to determine the interfacial bonding behavior of SHA coating to these metals and the ability of these metals to function as an electrode to deactivate SHA (Fig. S9). Among the tested metals, SHA exhibited the highest virgin *W*_adh_ value when contacting Ti and the lowest value when contacting Al. This result corroborated well with previously reported density functional theory (DFT) calculations where SHAM's binding energy with Ti (−200 kJ mol^−1^) was much higher when compared to that with Al (−2 kJ mol^−1^).^40,41^ The strong adhesion between SHAM and Ti likely arises from the robust bidentate adsorption to the surface oxide,^42^ whereas the heterogeneous oxide layer on stainless steel may reduce the uniformity and strength of SHAM coordination.^43^ After applying 1 V for 30 seconds, the determined *W*_adh_ values of SHA decreased to around 0.3–0.4 J m^−2^, a reduction of 81%, 72%, and 39% for Ti, SS, and Al, respectively. The different metals used here are highly electrically conductive for serving as an electrode (2.52, 1.36, and 37.7 MS m^−1^ for Ti, SS, and Al, respectively),^44^ making bulk conductivity unlikely to govern their electrochemical response. The measured *W*_adh_ values after exposure to applied electricity may be due to nonspecific interactions such as van der Waals forces, interfacial hydration, and contribution from incomplete removal of interfacial bonding,^45^ which may be the reason for similar *W*_adh_ values after electrical stimulation.

**Fig. 5:**
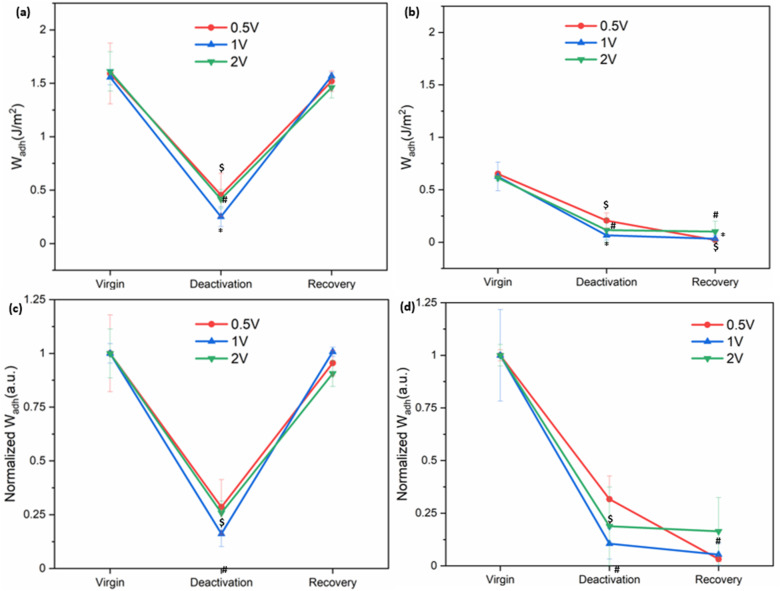
*W*_adh_ of (a) SHA and (b) CAT coating after deactivation with 0.5–2 V for 30 s and recovery through incubation in pH 5 buffer for 30 min. (c and d) *W*_adh_ values normalized to their average virgin adhesion values for SHA and CAT, respectively. ^$, *, #^*p* < 0.05 when compared to the virgin adhesive properties for 0.5, 1, and 2 V, respectively.

To evaluate whether electrochemically deactivated adhesives could recover their original adhesive properties, samples were incubated in pH 5 buffer for 30 minutes following deactivation (Fig. 5 and S10). Since 0.1 V did not induce measurable deactivation, recovery experiments focused on samples that were exposed to 0.5–2 V. After 30 seconds of exposure to applied electricity, *W*_adh_ values for SHA decreased by 71–84% and fully recovered its initial adhesive properties after incubation at pH 5. By contrast, CAT displayed a decrease in the *W*_adh_ value after exposure to the applied voltage but did not recover. Instead, *W*_adh_ values further declined in the third contact cycle (84–97% decrease from the virgin adhesive). This indicated that catechol irreversibly oxidized.

In addition to recovery by incubation at pH 5, we attempted a more convenient method by reversing the polarity of the applied potential.^20,46^ As shown in Fig. S11, the initial *W*_adh_ value was 1.66 ± 0.24 J m^−2^. After deactivation by applying 2 V for 30 s, adhesion decreased by 68%. Reversing the polarity by using Ti as the anode and Al as the cathode and applying 2 V for an additional 30 s did not restore the original *W*_adh_ value. The failure to recover adhesion may be attributed to continued electron transfer under prolonged charging, which could induce isomerization of the SHAM chelate ligand and disrupt coordination with Ti, thereby preventing adhesion recovery.^47,48^

Video S1 demonstrates the deactivation and recovery of SHA. Initially, the SHA-coated Al disc adhered to the Ti hemisphere in the presence of electrolyte, confirming adhesive properties under wet conditions. When the power supply was activated and the Ti electrode was connected as the cathode, application of 2 V caused the Al disc to detach within 2 seconds due to the loss of adhesion. The adhesive remained deactivated despite multiple contacts with the Ti hemisphere. After incubating in a pH 5 buffer for 30 minutes, the Ti hemisphere was once again able to lift the Al disc, confirming full recovery of adhesion.

Litmus papers were used to track the changes in pH of the interfacial solution as a result of electrical stimulation (Fig. S12). Application of 1 V for 30 s increased the pH at the interface from 7.4 to 9–10. The applied voltage generated a locally basic environment through water electrolysis.^49^ This result indicated that the changes in pH contributed to adhesive deactivation. UV-vis spectroscopy was utilized to further investigate the effect of pH change on the adhesive molecules. The copolymers contain hydrophobic side chains (*e.g.*, PyMA) and are poorly water soluble. As such, UV-vis spectra of DHMAAB and DMA were measured with sequentially changing the pH of the solution.

As shown in Fig. S13a, DHMAAB displayed absorption peaks at 275 and 314 nm, characteristic of protonated SHAM in its initial state.^22^ When the pH was increased to ∼9, the absorbance at 314 nm decreased, while a new band at 334 nm emerged, indicative of the doubly deprotonated form of SHAM. This shift reflected the alkalinization-induced deprotonation, which disrupted the metal–ligand chelation and thereby reduced adhesion. Returning the solution to pH 5 restored the protonated form, re-establishing chelation and confirming a fully reversible adhesion cycle. The concomitant decrease in absorbance at 280 nm may be associated with dilution of DHMAAB. In contrast, catechol exhibited a single peak at 275 nm under neutral conditions (Fig. S13b). Upon increasing the pH to ∼9, its absorbance at 275 nm intensified, while a broad band appeared between 400 and 600 nm, consistent with quinone formation.^50,51^ This oxidation accounted for the irreversible adhesion loss observed in CAT. Notably, the quinone band persisted after returning the solution to pH 5, confirming that catechol oxidation is largely irreversible and prevents adhesion recovery.

**Fig. 6:**
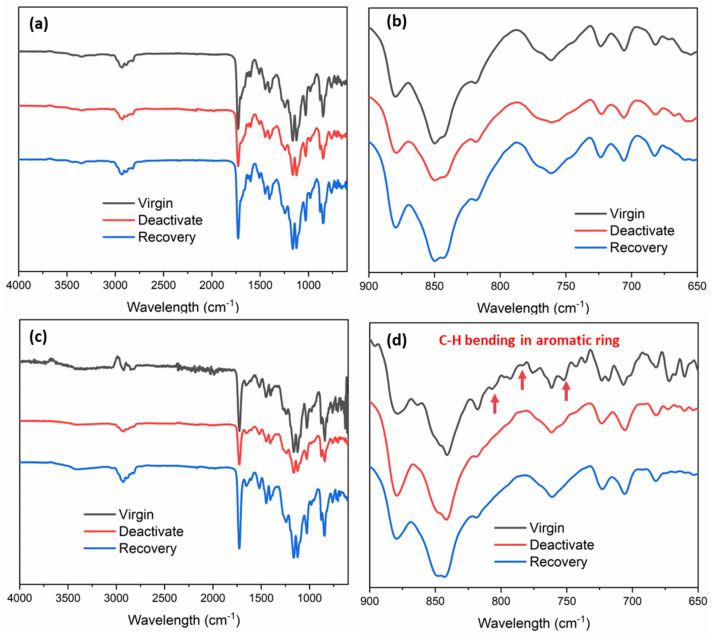
FTIR spectra of (a and b) SHA and (c and d) CAT coatings after deactivation under 1 V potential for 30 s and recovery in a pH 5 buffer for 30 min. The red arrows in (d) indicate the oxidation of catechol.

FTIR was employed to verify the molecular structures of SHA and CAT coatings before and after electrochemical deactivation and recovery treatments (Fig. 6). Both copolymers exhibited characteristic signals of the MEA backbone (C=O at 1728 cm^−1^) and aromatic ring associated with SHAM, catechol, or pyrene (C=C at 1448 cm^−1^ and 1512 cm^−1^). In addition, the hydroxamic acid side chain in SHA was identified by the N–O stretching band at 1599 cm^−1^, distinguishing SHA from CAT. For SHA, the FTIR spectra showed negligible changes in peak positions between the virgin, deactivated, and recovered states, indicating structural stability under the applied treatments. In contrast, CAT exhibited clear spectral changes following deactivation. As shown in Fig. 6d, the characteristic C–H bending peaks of catechol's aromatic ring (806, 776, and 752 cm^−1^) disappeared after charging at 1 V for 30 seconds and remained absent after attempted recovery. These results indicated irreversible oxidation of the aromatic ring to quinone, consistent with the observed loss of adhesion and failure to regain adhesive functionality.

**Fig. 7:**
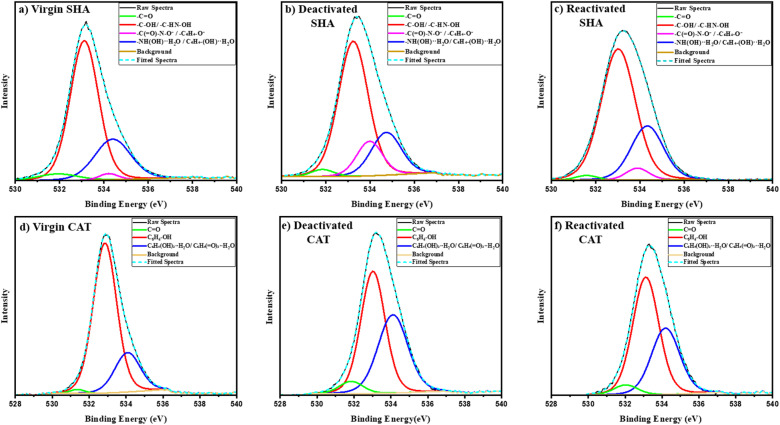
High-resolution O 1s XPS spectra of the SHA (a–c) and CAT (d–f) coatings in virgin (a and d), electrochemically deactivated (b and e), and reactivated states (c and f) featuring peak deconvolution.

X-ray photoelectron spectroscopy (XPS) was conducted to analyze the chemical changes in the adhesive coatings after electrochemical treatment and acidic recovery. The survey spectra indicated the presence of carbon, oxygen, and nitrogen (Fig. S14). High-resolution C 1s spectra displayed minor variations (Fig. S15, Tables S3 and S4), but interpretation was complicated by the predominant C–C peak (∼284.7 eV) resulting from overlapping contributions of the polymer backbone. The O 1s spectra showed distinct shifts related to SHAM and catechol oxygen functionalities (Fig. 7, Tables S5 and S6), offering direct insight into interfacial chemical changes. Four oxygen deconvoluted peaks were identified for SHA: hydroxamate carbonyl oxygen in –C(=O)–NHOH at 531.6–531.9 eV (1.5–2.5%, stable throughout), protonated hydroxamate (–C(=O)–N–**O**H) and phenolic hydroxyl groups (C_6_H_4_–**O**H) at 533–533.3 eV (68.23% in virgin),^52^ high binding energy component assigned to deprotonated or weakly coordinated oxygen species including C(=O)–N–**O**^−^ and C_6_H_4_–**O**^−^ at 533.8–534.2 eV (4.02% in virgin)^52^ and hydrogen-bonded interfacial oxygen associated with absorbed H_2_O bonded to hydroxamate and phenol groups (534–535 eV; 25.22% in virgin).^53^ After electrochemical deactivation, the protonated hydroxamate/phenolic component reduced from 68.23% to 63.23%, which coincided with an increase in the deprotonated oxygen fraction from 4.02% to 14.10%. Upon reactivation in an acidic buffer, the protonated hydroxamate/phenolic oxygen recovered to 68.05%, while the deprotonated oxygen reduced to the original 4.05%. These shifts in oxygen signals indicated the reversible nature of SHAM as a result of deprotonation and reprotonation of SHAM's hydroxinamic acid and phenol hydroxyl groups (C(=O)–N–OH → C(=O)–N–O^−^ → C(=O)–N–OH; C_6_H_4_–OH → C_6_H_4_–O^−^ → C_6_H_4_–OH) without irreversible oxidation. The hydrogen-bonded oxygen component (534.3–534.7 eV) decreased from 25.22% to 20.15% after deactivation, likely due to a reduction in hydrogen bonding following the deprotonation of the –OH groups. It increased back to 26.34% when reactivated, close to the virgin value, showing that the hydrogen bond at the surface had recovered.^54^

Conversely, CAT was irreversibly oxidized, as demonstrated by a decrease in the phenolic hydroxyl oxygen signal (C_6_H_4_(**O**H)_2_; 532.8–533.1 eV)^55^ from 75.00% to 35.95% as a result of electrical stimulation and a lack of significant recovery when CAT was treated with an acidic buffer (38.11%). Coincidentally, the quinone signal (C_6_H_4_(=**O**)_2_; 531.3–531.7 eV)^56,57^ increased from 1.24% to 5.14% and only reduced slightly to 4.63%. The high energy oxygen peak at 534.0–534.5 eV is associated with phenolic hydroxyl or quinoid carbonyl groups bonded with water (C_6_H_4_(**O**H)_2_⋯H–O–H and C_6_H_4_(**O**H)_2_⋯H–O–H).^53,54^ The high-binding-energy oxygen increased from 21.94% to 41.70% and remained at 36.19% after acid treatment. This result suggests a rise in surface polarity and a stronger interaction between quinonoid carbonyl groups and surface moisture or adsorbed H_2_O.^58^ XPS spectra clearly indicated that catethol irreversibly oxidized to its poorly adhesive quinone form after electrical stimulation.

**Fig. 8:**
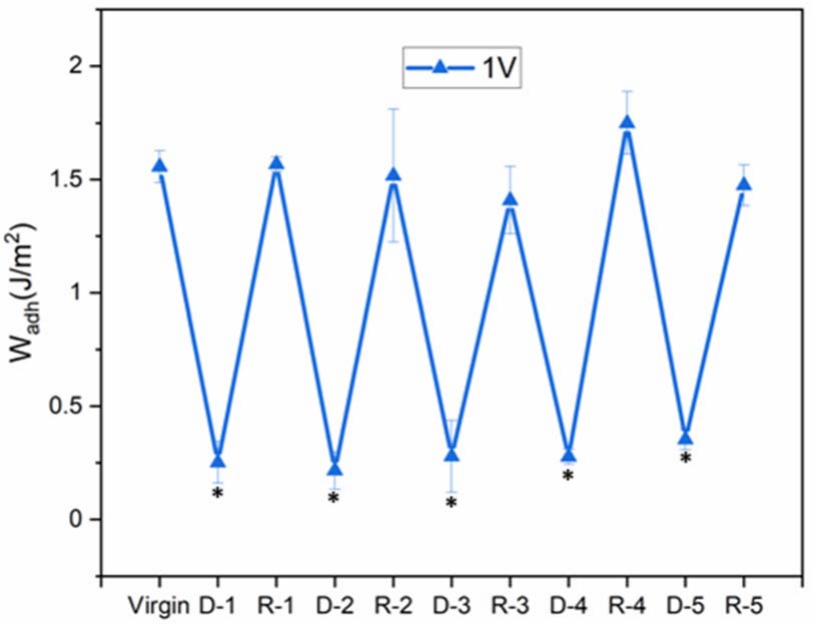
*W*_adh_ of SHA tested in successive contact cycles with the application of 1 V for 30 s (denoted as D) for the reduction of adhesive properties and incubation in a pH 5 buffer for 30 min for adhesion recovery (denoted as *R*). Numerical values 1–5 represent the cycle number. *p < 0.05 when compared to the virgin adhesive properties.

Finally, the reversibility of the SHAM-based adhesive was evaluated through repeated deactivation–recovery cycles. The coating was subjected to 1 V for 30 seconds, followed by 30 minutes of incubation in a pH 5 buffer. After each deactivation step, *W*_adh_ values decreased by 80–86% but consistently returned to the original value after recovery (Fig. 8). No significant differences were observed compared with the initial adhesion performance. Up to five successful cycles were completed, confirming the reversible and repeatable electro-responsive behavior of the SHAM-based adhesive.

In summary, SHAM demonstrated strong potential as an electrochemically controlled, switchable adhesive for metallic surfaces under wet conditions. CV and EIS results confirmed that SHA and CAT possess comparable electrical resistance, indicating that the observed differences in adhesion arose from the intrinsic electrochemical behavior of the adhesive monomers. In its protonated state, SHAM binds to the Ti surface primarily through the hydroxamate functional group, which is a strong chelator of metals.^24,25^ On Ti substrates, the hydroxamate group can coordinate through bidentate or bridging interactions involving the carbonyl oxygen and the N–O moiety, resulting in strong interfacial adhesion. Upon electrochemical charging, local alkalinization near the cathode leads to deprotonation of the hydroxamic acid. Deprotonation alters both the charge state and coordination geometry of the hydroxamate group, which weakens its affinity for Ti by disrupting optimal chelation and reducing the stability of the metal–ligand complex. This change in coordination is consistent with the observed loss of adhesion during deactivation. Subsequent exposure to acidic conditions restores protonation of SHAM, re-establishing metal–ligand interactions and enabling recovery of adhesion.

SHAM underwent reversible deprotonation upon charging, followed by protonation in acidic media to restore strong adhesion. In contrast, catechol was irreversibly oxidized to quinone, preventing reduction and recovery under pH 5 conditions. Both adhesives can be deactivated using electrical potential as low as 0.5 V, which is lower than the electrolytic voltage (1.23 V) of water.^59^ This low activation threshold is advantageous, as it prevents gas bubble formation, minimizes electrode corrosion, and reduces harmful byproducts such as hydrogen peroxide that accelerate metal degradation.^60–64^ A key distinction between the two adhesive moieties lies in their reversibility. SHAM fully recovered its adhesive properties after electrical stimulation, whereas catechol did not. It was previously demonstrated that catechol requires a temporary protecting group in the form of boronic acid to recover its initial adhesive properties after electrochemical oxidation.^20,46^ This highlights SHAM's superior chemical stability to that of catechol and its potential in functioning as a switchable adhesive molecule that is responsive to electrochemical control.

We compared the performance of SHAM with representative electricity responsive reversible adhesives reported in the literature (Table S7). When a protecting group in the form of phenylboronic acid is incorporated into a catechol-containing adhesive, the adhesive system demonstrated responsiveness to the applied voltage in the range of 1–2 V with equivalent adhesive performance.^21^ However, this adhesive demonstrated a higher switching ratio due to the formation of a boronate complex to completely deactivate catechol adhesion. Electroadhesives consisting of polyelectrolytes demonstrated high reversibility.^65,66^ However, the adhesive requires applied electricity to generate electrostatic interaction and is limited to bonding between ionic surfaces. Thermal responsive adhesives can be modified to respond to applied electricity through a electrothermal heating effect, but these adhesives often suffer from a long transition time.^67^ SHAM reported here presents a new, alternative adhesive molecule for designing electroresponsive adhesives.

This study demonstrates that SHAM-based coatings can be directly deactivated on conductive substrates, with the substrate serving both as an electrode and as the adhesive contact surface. While this approach is effective, it restricts the use for contacting nonconductive surfaces, and a better integration of the counter electrode into the contacting surface may be required.^20^ In the present study, reactivation required acidic incubation, which is not a practical trigger for real-world applications. We attempted to achieve recovery by reversing the current direction. However, it was unsuccessful, likely due to ligand (SHAM) isomerization or continued electron transfer-induced chelate rearrangements, which disrupted SHAM–Ti binding.^47,48^ Future work will focus on confining the electrochemical reactions within the adhesive circuit itself, enabling reversible adhesion control on a broader range of conductive and insulating substrates.

Despite these limitations, SHAM offers significant advantages over catechol, including stronger initial adhesion, robust reversibility without the need for protective groups, and stability under basic conditions. These findings position SHAM as a promising adhesive molecule for designing electro-responsive, switchable adhesives for contacting wetted surfaces. Its tunable, low-voltage operation and repeatable reversibility open pathways for practical applications in reusable biomedical adhesives, minimally invasive wound dressings, wearable electronics, and underwater robotic manipulators, where precise and reversible adhesion is essential.

## 4. Conclusion

SHAM-containing adhesives exhibit electro-responsive and switchable interfacial bonding to metallic surfaces under wet conditions without the need for protecting groups. Adhesion could be effectively deactivated at voltages as low as 0.5 V within 30 seconds. Upon exposure to a mildly acidic pH environment, SHAM fully regained its initial adhesive strength owing to the reversible protonation–deprotonation reaction. In contrast, catechol-based adhesives suffered irreversible adhesion loss due to oxidative conversion to quinone, underscoring SHAM's superior chemical stability and reversibility. Its low-voltage activation, strong adhesion, and full reversibility make SHAM a promising candidate for reusable biomedical adhesives, wound dressings, wearable electronics, and underwater robotics requiring precise on-demand adhesion.

## Author contributions

K. W. contributed to conceptualization, visualization, and project administration. K. W., V. K., A. A. D., and F. R. contributed to methodology, investigation and formal analysis. B. P. L. contributed to conceptualization, supervision, and funding acquisition. All authors contributed to writing and editing of the manuscript.

## Conflicts of interest

The authors declare no competing financial interest.

## Supplementary Material

SC-OLF-D5SC07881D-s001

SC-OLF-D5SC07881D-s002

## Data Availability

The data supporting this article have been included as part of the supplementary information (SI). Supplementary information: Tables S1–S6 and Fig. S1–S15 displaying GPC characterization, XPS spectra, NMR spectra, FE-SEM characterization, EIS spectra of two copolymers, UV-vis spectra of two monomers, and representative JKR contact curves of adhesive coatings influenced by electricity deactivation and pH driven reactivation; deactivation–recovery cycling of an electro-responsive SHAM adhesive (Video S1). See DOI: https://doi.org/10.1039/d5sc07881d.
