# Supplementary material for: Salicylhydroxamic acid as an electro-responsive and switchable adhesive molecule
Source: Chem Sci. 2026 Apr 2;17(20):10113–25. doi: 10.1039/d5sc07881d (PMC13066939; doi:10.1039/d5sc07881d)
Supplement: SC-017-D5SC07881D-s002 [file SC-017-D5SC07881D-s002.pdf]

Supplementary Information (SI) for Chemical Science.
This journal is © The Royal Society of Chemistry 2026

Supporting Information

Salicylhydroxamic acid as an electro-responsive and switchable adhesive molecule

*Kan Wang, Vedika Khare, Abhilash Arjan Das, Seyedehfatemeh Razaviamri, and Bruce P. Lee**

Department of Biomedical Engineering, Michigan Technological University, 1400 Townsend Drive, Houghton, Michigan, 49931, United States

* Correspondence: Bruce P. Lee,
E-mail: bplee@mtu.edu

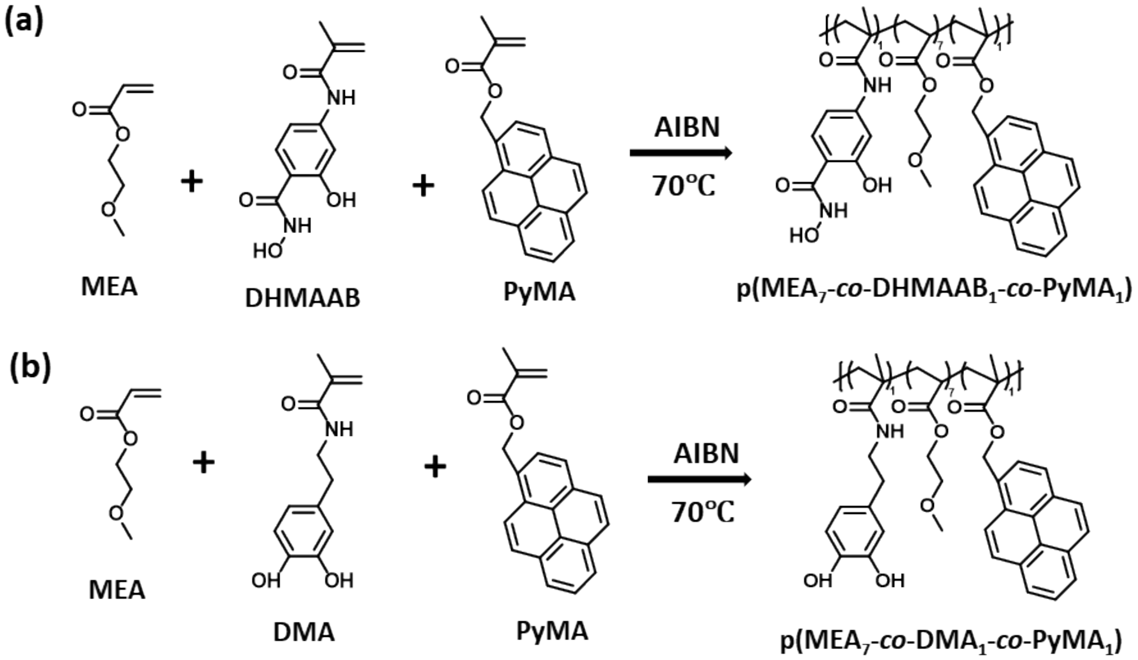

Figure S1. Thermo-initiated polymerization to prepare the adhesive copolymers SHA and CAT.

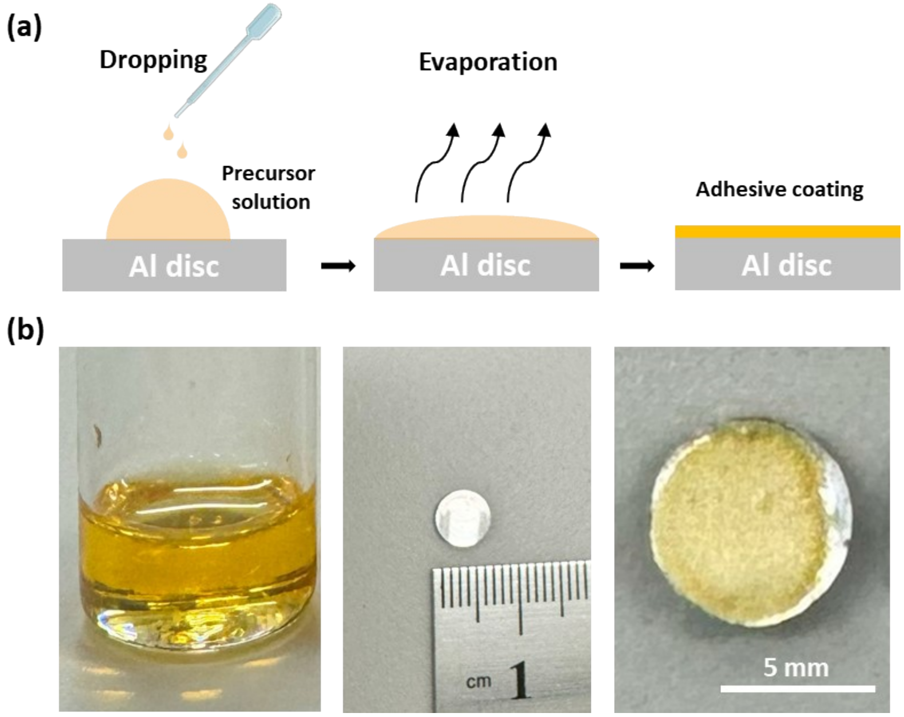

Figure S2. (a) Schematic diagram of the drop casting method. (b)Photographs of the adhesive precursor solution (left), Aluminum (Al) disc (middle), and adhesive-coated Al disc (right).

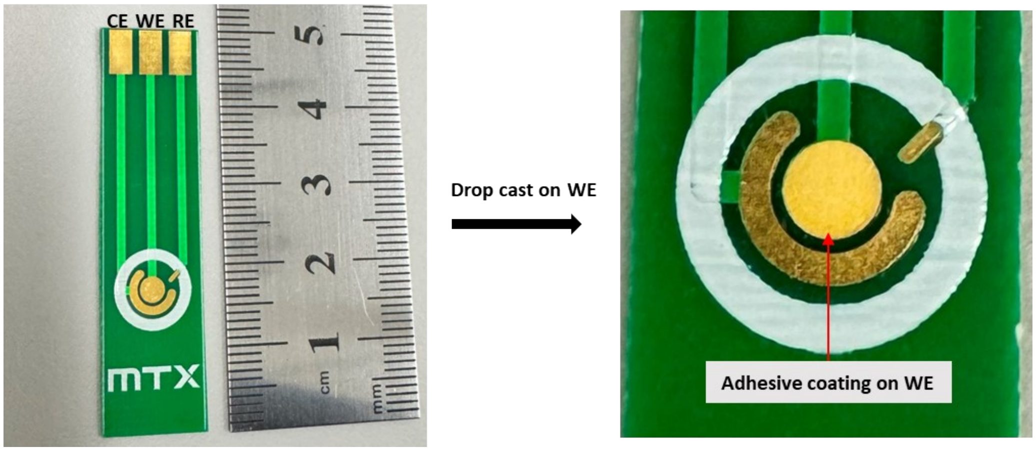

Figure S3. Images of the IDEs (left) and the adhesive-coated WE (right). Area of WE = 7 mm^2.

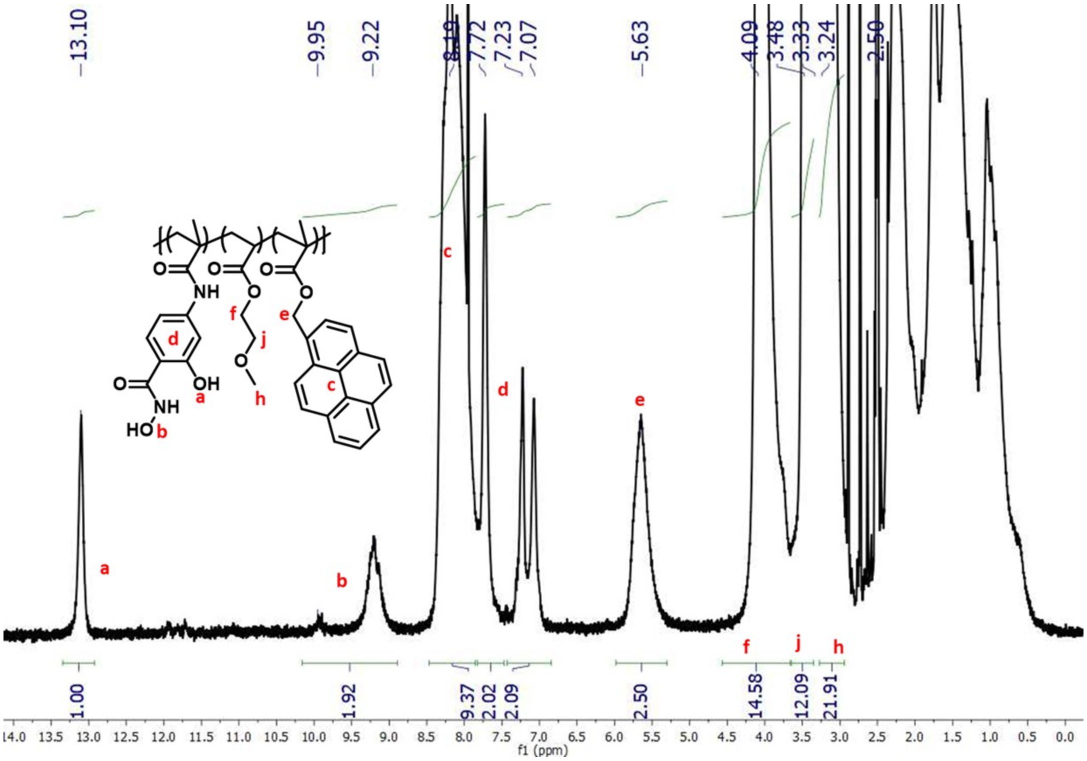

Figure S4. ^{1}H NMR spectra of SHA.

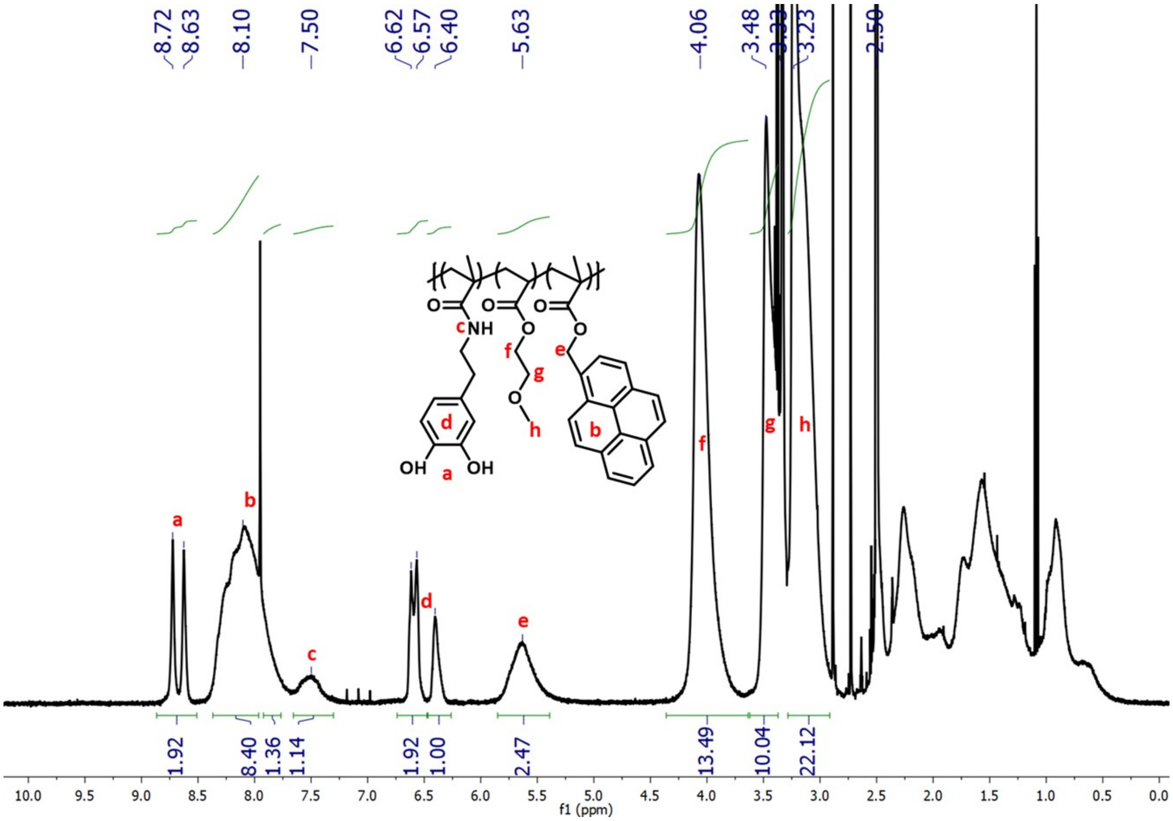

Figure S5. ^{1}H NMR spectra of CAT.

Table S1. Molecular weights of polymers based on GPC analysis.

Polymer	$\bar{M}_n$ (g/mol)	$\bar{M}_w$ (g/mol)	PDI
SHA	2.17×10^5	1.13×10^6	5.19
CAT	1.63×10^4	3.50×10^4	2.14

Table S2. Redox peaks of two polymers based on CV analysis.

Polymer	A1 (V)	C1(V)	C2(V)
SHA	+0.54	-0.31	-0.98
CAT	+0.95	-0.22	-0.85

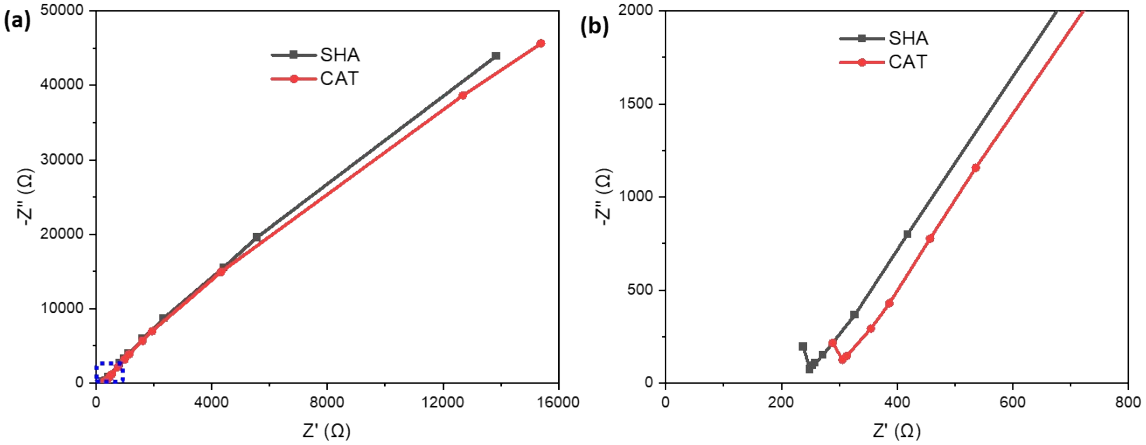

Figure S6. Nyquist plot of SHA and CAT. Panel (b) is a zoomed in plot from (a).

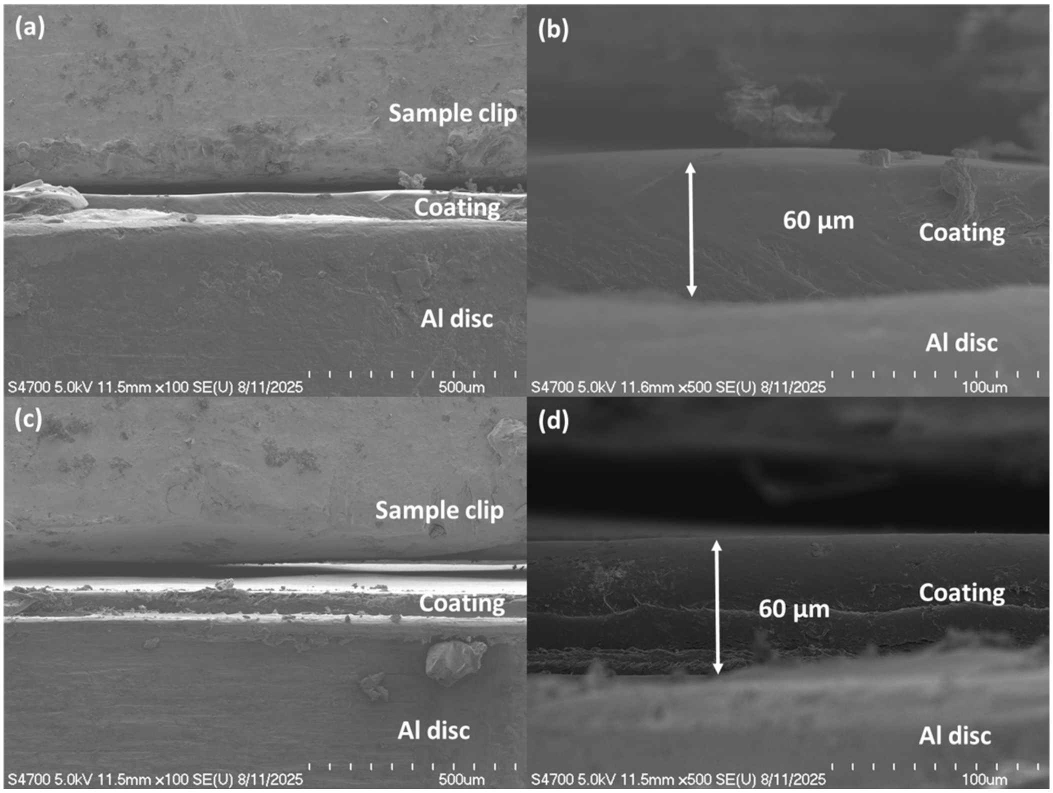

Figure S7. FE-SEM images of cross-section view of (a)(b) SHA-coated and (c)(d) CAT-coated Al disc.

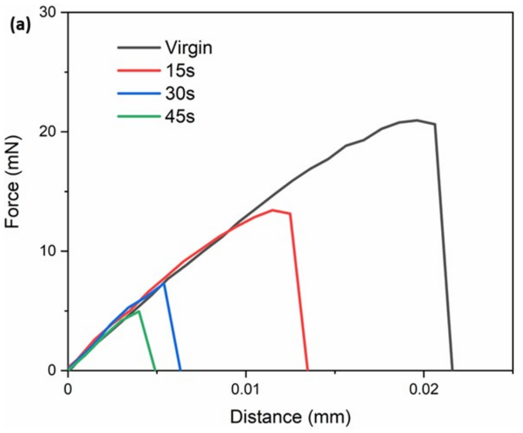

Figure S8. Typical JKR contact curves of (a) SHA and (b) CAT adhesive coatings with the application of 0.5V for 0s, 15s, 30s, and 45s. Voltage was applied using a Ti hemisphere and an Al disc as electrodes.

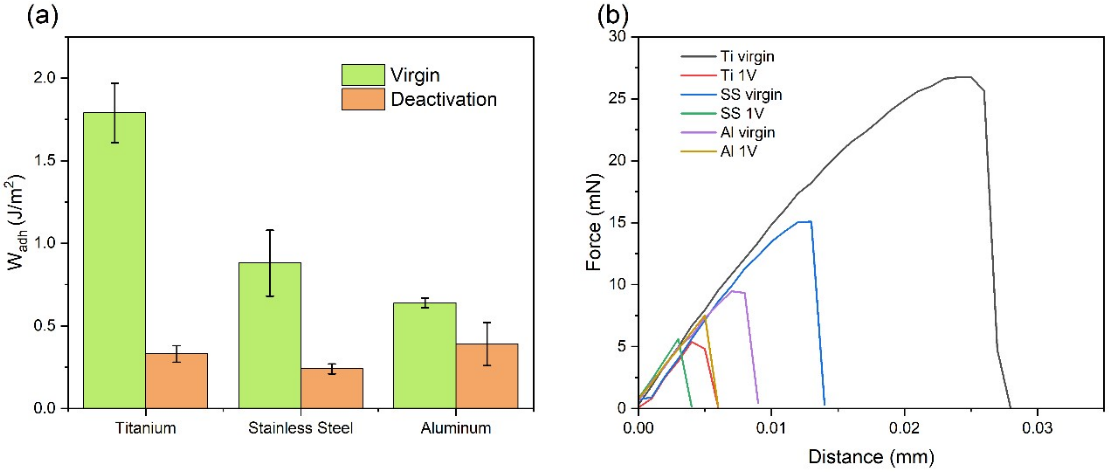

Figure S9. (a) W_{adh} and (b) JKR contact curves of SHA coating contacting different metal substrates before and after deactivation by 1V of applied potential for 30 seconds.

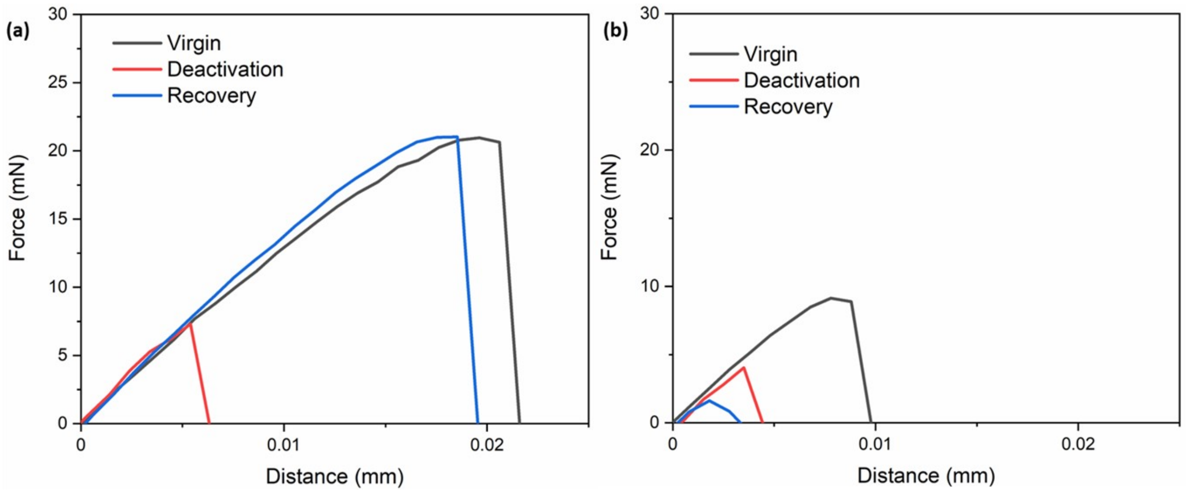

Figure S10. Representative JKR contact curves of (a) SHA and (b) CAT. SHAM-based adhesive showed recovered adhesive properties after application of 0.5V for 30s. Each deactivated sample was incubated in pH 5 for 30 mins to recover adhesive properties.

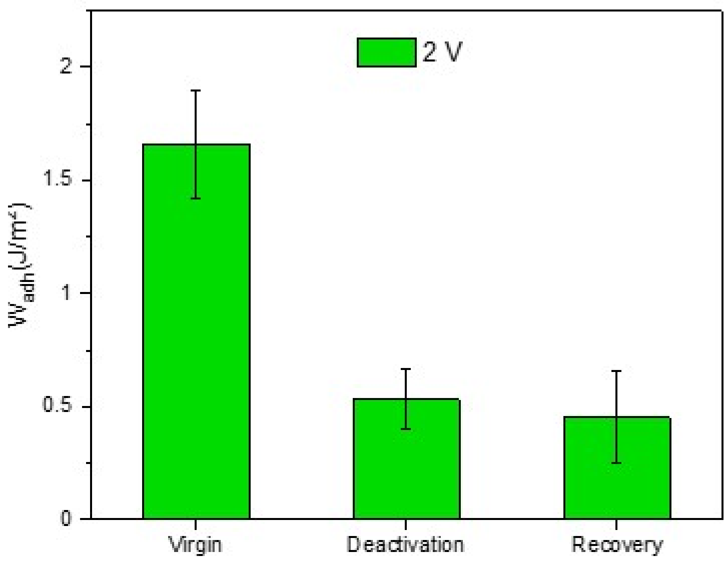

Figure S11. W_{adh} of SHA coating after deactivation at 2 V for 30 s and attempted recovery by reversing polarity and charging at 2 V for 30 s.

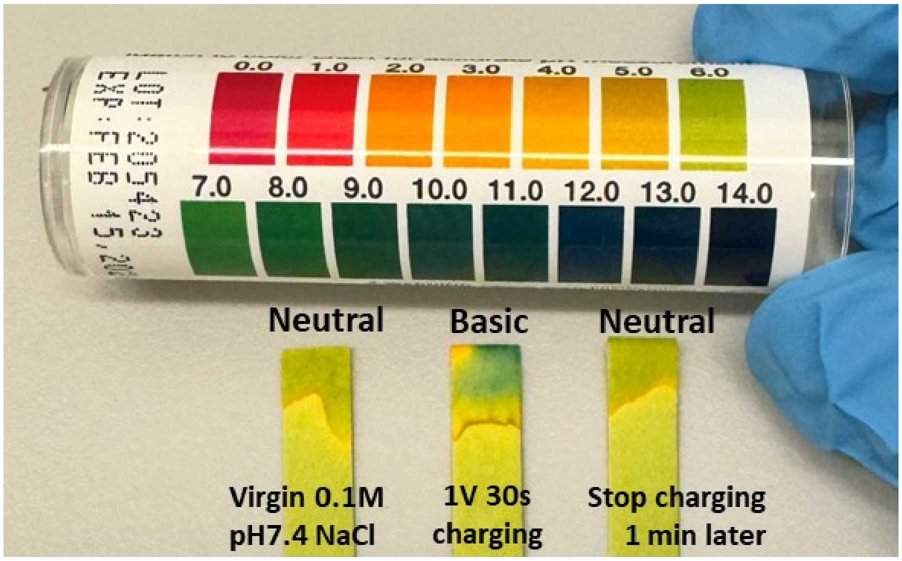

Figure S12. Photographs of litmus paper to track the changes in pH of the solution at the interface prior to application of electricity, after exposing to 1V for 30s, and 1 min after the exposure of applied electricity.

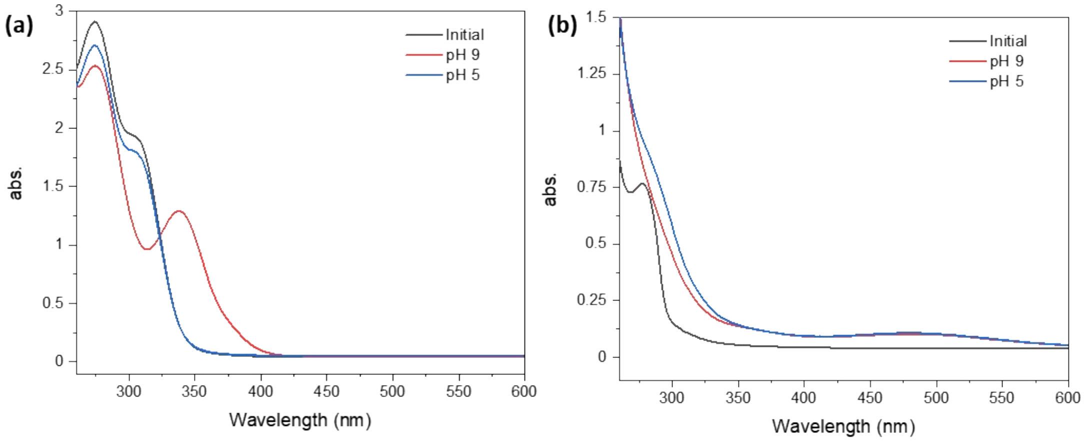

Figure S13. UV-vis spectra of 0.2 mM of (a) DHMAAB and (b) DMA measured before sequentially changing solution pH (initial measurement at pH 7.4), and after changing the pH to 9 and then 5. The pH was adjusted by dropwise addition of pH 14 NaCl solution and pH 0 HCl solution, respectively.

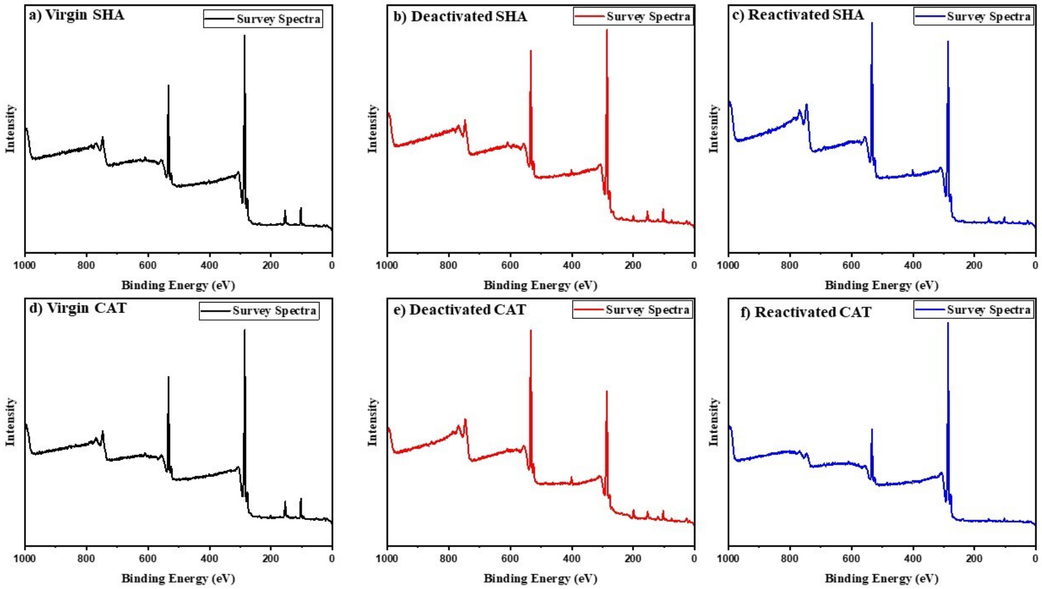

Figure S14. XPS survey spectra of the SHA (a-c) and CAT (d-f) adhesive coatings under virgin, electrochemically deactivated, and reactivated conditions show the presence of C, O, and N elements across all surface states.

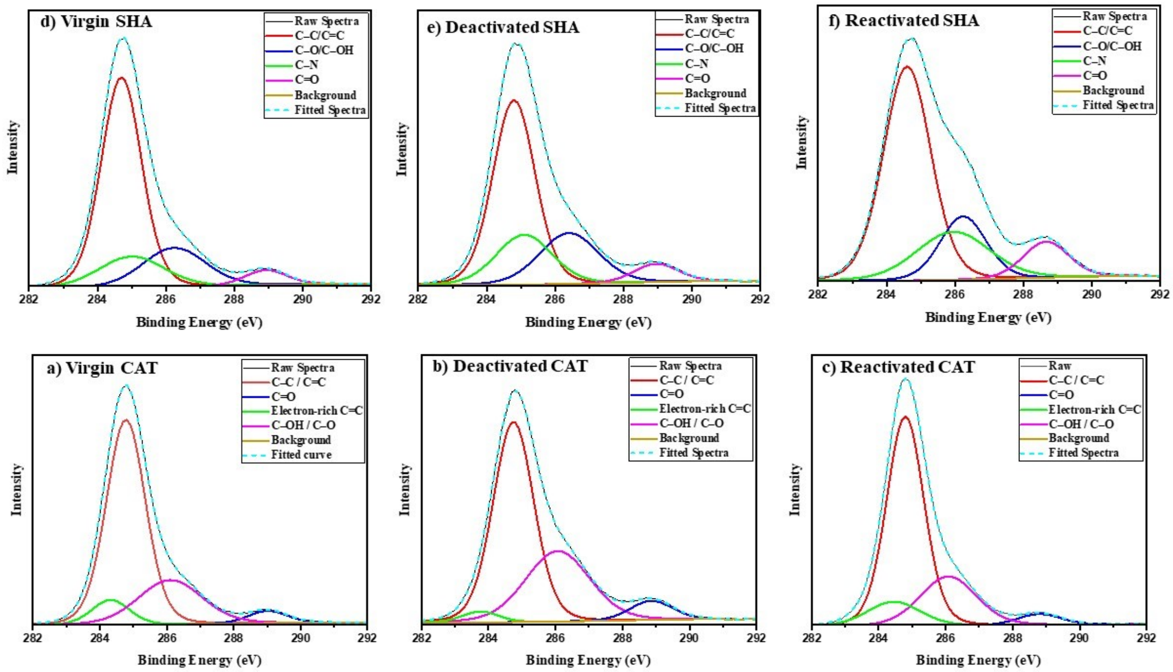

Figure S15. High- resolution C 1s XPS spectra of the SHA (a-c) and CAT (d-f) coatings in virgin (a,d) ,electrochemically deactivated (b,e) , and reactivated states (c,f) , featuring peak deconvolution into C-OH/C-O, C=O, absorbed oxygen, and transitory oxygen components.

Table S3. Summary of high-resolution C 1s XPS spectral deconvolution detailing peak positions and relative area percentages for SHA coatings in virgin, deactivated, and reactivated phases

**Assigned Carbon C 1s**	**C–C/C=C**	**C–N**	**C–O/C–OH**	**C=O**
		**Virgin**		
**Binding Energy (eV)**	284.70	285.00	286.24	288.9
**% Area**	62.26	14.99	18.12	4.63
		**Deactivated**		
**Binding Energy (eV)**	284.8	285.07	286.38	288.9
**% Area**	53.83	19.01	21.53	5.63
		**Reactivated**		
**Binding Energy (eV)**	284.6	285.94	286.2	288.6
**% Area**	60.67	19.36	16.00	3.97

Table S4. Summary of high-resolution C 1s XPS spectral deconvolution detailing peak positions and relative area percentages for CAT coatings in virgin, deactivated, and reactivated phases

**Assigned Carbon C 1s**	**Electron-rich C=C**	**C–C / C=C**	**C–OH / C–O**	**C=O**
		**Virgin**		
**Binding Energy (eV)**	284.63	284.78	286.09	288.97
**% Area**	9.30	65.53	23.05	4.13
		**Deactivated**		
**Binding Energy (eV)**	283.76	284.74	286.06	288.86
**% Area**	2.79	58.74	32.65	5.82
		**Reactivated**		
**Binding Energy (eV)**	284.45	284.79	286.07	288.79
**% Area**	7.62	63.30	22.44	3.80

Table S5. Summary of high-resolution O1s XPS spectral deconvolution detailing peak positions and relative area percentages for SHA coatings in virgin, deactivated, and reactivated phases

Assigned Oxygen (O 1s)	-C(=O)-NHOH	-C(=O)-N-OH / C_6H_4-OH	-C(=O)-N-O^- / C_6H_4-O^-	C_6H_4-OH··HO-H_2O / -C(=O)-N-OH···H_2O
		**Virgin**		
**Binding Energy (eV)**	531.94	533.10	534.20	534.39
**% Area**	2.53	68.23	4.02	25.22
		**Deactivated**		
**Binding Energy (eV)**	531.84	533.23	533.90	534.73
**% Area**	2.52	63.23	14.10	20.15
		**Reactivated**		
**Binding Energy (eV)**	531.58	533.01	533.81	534.30
**% Area**	1.55	68.05	4.05	26.34

Table S6. Summary of high-resolution O1s XPS spectral deconvolution detailing peak positions and relative area percentages for SHA coatings in virgin, deactivated, and reactivated phases

Assigned Oxygen (O 1s)	$C_6H_4(=O)_2$	$C_6H_4(OH)_2$	$C_6H_4(OH)_2$··H_2O/ $C_6H_4(=O)_2$··H_2O
		**Virgin**	
**Binding Energy (eV)**	531.36	532.84	534.00
**% Area**	1.24	76.82	21.94
		**Deactivated**	
**Binding Energy (eV)**	531.74	533.02	534.30
**% Area**	5.14	53.15	41.70
		**Reactivated**	
**Binding Energy (eV)**	531.36	533.13	534.32
**% Area**	4.65	59.16	36.19

Table S7. Comparison of reversible adhesive systems.

Mechanism of Switching	Adhesive System	Level of Applied Electricity	Work of Adhesion (J/m^2)	Switching Ratio*	Reversible cycles	Ref.
Electrochemical	SHAM-based adhesive	0.5-2 V	1.6	5-7	5	This Work
	Catechol with Phenylboronic acid	1-2 V	1.2	3-20	5	1
Electricity induced electrostatic interaction	Polyelectrolyte-based adhesive	1-2V	2.2	3	30	2
		10V	-	15-25	10	3
Electrothermal Responsive	Hydrogen bonding-based adhesive	4-8V	-	1.1-1.3	2	4

* Switching ratio is defined as the ratio between the maximum and minimum adhesion values.

References:

1. M. S. A. Bhuiyan, B. Liu, J. Manuel, B. Zhao and B. P. Lee, Effect of Conductivity on In Situ Deactivation of Catechol–Boronate Complexation-Based Reversible Smart Adhesive, *Biomacromol.*, 2021, **22**, 4004-4015.
2. H. J. Kim, L. Paquin, C. W. Barney, S. So, B. Chen, Z. Suo, A. J. Crosby and R. C. Hayward, Low-Voltage Reversible Electroadhesion of Ionoelastomer Junctions, *Adv. Mat.*, 2020, **32**, 2000600.
3. S. Lu, Z. Ma, M. Ding, Y. Wu, Y. Chen, M. Dong and L. Qin, Reversible electroadhesion induced through low ion concentration migration for biomedical applications, *Chemical Engineering Journal*, 2024, **486**, 150393.
4. Q. Deng, S. Han, Y. Wu, Y. Chen, Y. Zhang, Y. Zhao, S. Chen and J. Zhu, Robust and Reversible Thermal/Electro-Responsive Supramolecular Polymeric Adhesives via Synergistic Hydrogen-Bonds and Ionic Junctions, *Angew. Chem., Int. Ed.*, 2025, **64**, e202415386.
